# No robust evidence for an effect of head-movement propensity on central bias in head-constrained scene viewing, despite an effect on fixation duration

**DOI:** 10.1167/jov.25.4.10

**Published:** 2025-04-16

**Authors:** Patricia R. Mueller, Sabine Grimm, Wolfgang Einhäuser

**Affiliations:** 1 Physics of Cognition Group, Institute of Physics, Chemnitz University of Technology, Chemnitz, Germany; 2 Cognitive Systems Lab, Institute of Physics, Chemnitz University of Technology, Chemnitz, Germany; 3 Physics of Cognition Group, Institute of Physics, Chemnitz University of Technology, Chemnitz, Germany

**Keywords:** central bias, head-movement propensity, inter-individual differences, eye-movement, natural scene viewing

## Abstract

When viewing natural scenes, participants tend to direct their gaze towards the image center, the so-called “central bias.” Unless the head is fixed, gaze shifts to peripheral targets are accomplished by a combination of eye and head movements, with substantial individual differences in the propensity to use the head. We address the relation of central bias and head-movement propensity. In one part of the experiment, participants viewed natural scenes of two different sizes without moving their head. We found that the central bias of each individual scaled with image size. In another experimental part, the same participants stood in the center of a panoramic screen and shifted their gaze to peripheral targets. Target eccentricities were either instructed by text (endogenous mode) or by a bar appearing at the target location (exogenous mode). In this “peripheral-target” task, we found a strong correlation between the exogenous and the endogenous mode, indicating that they provide a robust measure of an individual's head-movement propensity. Despite substantial inter-individual variability in both tasks, no significant correlation was found between head-movement propensity and central bias, and a trend toward significance for a specific measure was brittle. However, individuals with a higher head-movement propensity tended to have shorter fixation durations in scene viewing. Our results suggest that central bias in free scene viewing on typical screen sizes is predominately determined by visual properties. Although head-movement propensity seems to affect some aspects of scene-viewing behavior (fixation durations), individual differences in central bias are not explained by head-movement propensity.

## Introduction

In natural scene viewing, participants tend to direct their gaze towards the image center (Clarke & Tatler, 2014; Ruddock, Wooding, & Mannan, 1995; Tatler, 2007; Tatler, Baddeley, & Gilchrist, 2005). Several explanations for this so-called “central bias” have been put forward, such as a tendency of “salient” image features to cluster in the center of photographs and a general benefit of a central viewing direction to reach all areas of an image with smaller saccades—but none explains the central bias in full (Tatler, 2007). The central bias was also found when presenting images off the screen center (Bindemann, 2010; Tseng, Carmi, Cameron, Munoz, & Itti, 2009) or placing the pre-trial fixation cross away from the screen center (Rothkegel, Trukenbrod, Schütt, Wichmann, & Engbert, 2017). Although it has been argued that the shape of the central bias in scene viewing is consistent across experimental settings (Clarke & Tatler, 2014), the extent of central bias varies considerably among individuals and among scenes (Nuthmann, Einhäuser, & Schütz, 2017), even within the same experiment. A form of central bias also persists when we move through the real world (Foulsham, Walker, & Kingstone, 2011; Ioannidou, Hermens, & Hodgson, 2016) but is less pronounced compared to viewing a comparable stimulus on a computer screen (’t Hart et al., 2009). These differences may be due to additional task demands in the real world (’t Hart & Einhäuser, 2012), which are especially present during walking (Kopiske, Koska, Baumann, Maiwald, & Einhäuser, 2021; Matthis, Yates, & Hayhoe, 2018), the limited projection surface of the screen in the laboratory setting, or the restriction of head and body movements in typical laboratory setups. When viewing natural scenes on a distant projection screen, whose angular subtense approximated that of monitors at typical viewing distances, observers’ central bias was smaller when they were standing freely than when they were sitting with their head constrained, especially during later phases of viewing (Backhaus & Engbert, 2024). Interestingly, this effect of posture was largely independent of an effect of task on central bias, which had also been found in an earlier study in standing observers (Backhaus, Engbert, Rothkegel, & Trukenbrod, 2020). Here, we ask a complementary question: do inter-individual differences in the usage of the head when standing freely and looking at peripheral targets (head-movement propensity) relate to differences in central bias during head-constrained scene viewing?

Unless the head is fixed, gaze shifts to peripheral targets are accomplished by a combination of eye and head movements. Aggregating the fields of both eyes while looking straight ahead, the human horizontal field of view is about 210 degrees of visual angle (dva) in total (Sidenmark & Gellersen, 2019). By moving the eyes (without moving the head) humans can reach additional ±50 to 55 dva horizontally (Guitton & Volle, 1987), but longer fixations of eccentricities larger than ±40 dva are uncomfortable for many participants (Fuller, 1992b). Incorporation of head movement eases gaze alignment to peripheral-targets. The head range is about ±80 to 90 dva along the horizontal axis (Sidenmark & Gellersen, 2019) but there are substantial inter-individual differences in the propensity to use the head for gaze-target-alignment (Fuller, 1992b; Stahl, 1999).

Here, we investigate if inter-individual differences in central bias and head-movement propensity are related. Specifically, we hypothesized that individuals with a larger tendency to move their head when they are free to do so, show a stronger central bias (i.e., a stronger tendency to look toward the center) in scene viewing with a constrained head. The rationale behind this hypothesis was the expectation that observers with higher head-movement propensity generally tend to use less of their oculomotor range and are therefore prone to look more centrally when the head constraint requires them to use their eyes instead. Nonetheless, we acknowledge that considering the results of Backhaus & Engbert (2024), where the possibility of head movements enlarges the central bias (i.e., reduces the average distance of gaze to image center), the reverse hypothesis might also have been plausible. In any case, all of our hypothesis testing is two-sided. In addition, we explore further parameters of scene viewing—in particular fixation duration and saccade amplitude—and their relationship with head-movement propensity.

## Methods

The experiment consisted of two parts: (i) a scene-viewing experiment conducted in a conventional laboratory eye-tracking setup with a monitor of typical size and fixed head position (“scene-viewing task”) and (ii) an experiment to measure head-movement propensity using a large panoramic screen and allowing participants to freely move head and upper body to look at peripheral targets (“peripheral-target task”). The two parts were conducted in direct succession on the same day with an about 15-minute break in-between, which was needed to walk from one laboratory to the other. The order of parts was counterbalanced, half of the participants started with the scene-viewing task, half with the peripheral-target task.

### Participants

Thirty-two individuals, 22 women and 10 men with an average age of 23.81 ± 4.43 years (range: 19 to 39 years), participated in the study. Data of two participants had to be excluded from the analysis of the peripheral-target task data due to a lack of data after trial-error exclusions (for the predefined criterion see “Data extraction, processing, and variables”), leaving us with a set of *N* = 30 participants for the comparison of central bias and head-movement propensity. For the analyses that pertain only to the scene-viewing data, we used the full set of *N* = 32. Sample size was defined before the experiment and based on the assumption of a large effect (*r* = 0.5), an alpha-level of 0.05 and a power of 80%. This yields a minimum sample size of 29; as counterbalancing constraints demanded the size to be divisible by 4, *N* = 32 was decided upon. Participants were recruited via a dedicated mailing list, had normal or corrected-to-normal vision and normal color vision as assessed by Ishihara plates. Before the experiment, all participants gave written, informed consent. Participants received either course credit or a monetary reimbursement of 8€/h. All procedures, including the sample size calculation, were approved by the Chemnitz University of Technology ethics committee (case-no: 101531269).

### Apparatus

#### Conventional laboratory setup

In the scene-viewing part of the experiment stimuli were presented centrally on a VIEWPixx/3D monitor (VPixx Technologies Inc., Saint-Bruno, QC, Canada) running at a frame rate of 120 Hz and a resolution of 1920 × 1080 px. The non-image background was set to mid-level gray of 49.8 cd/m^2^. Participants sat 0.57 m away from the screen while their head was stabilized by a chin and forehead rest. We measured monocular gaze position at 1000 Hz sampling rate with the Eyelink-1000 infrared camera system (SR Research, Kanata, ON, Canada) positioned in tower-mount configuration. For stimulus preparation, presentation and eye-tracker control MATLAB (The MathWorks, Natick, MA, version R2016a) was used, including the Psychophysics Toolbox (Brainard, 1997; Kleiner, Brainard, & Pelli, 2007), which incorporates Eyelink Toolbox extensions (Cornelissen, Peters, & Palmer, 2002).

#### Panoramic-screen setup

**Figure 1.**
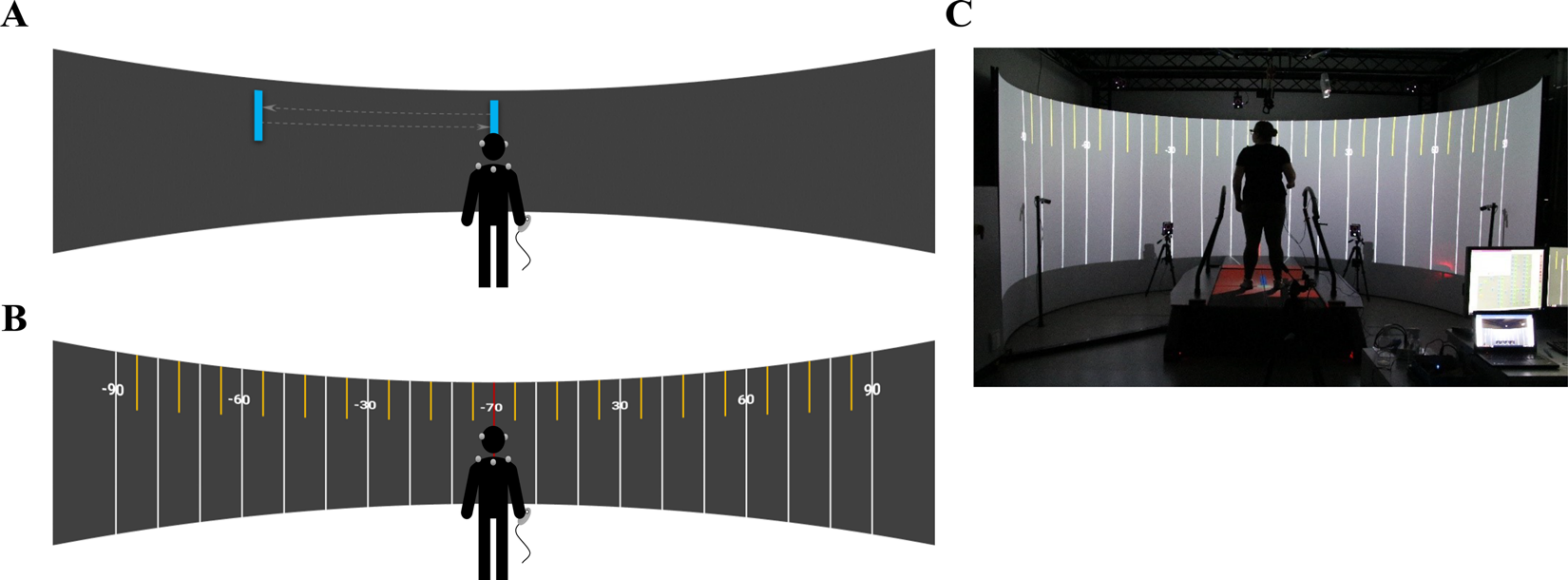
Panoramic setup and presentation modes of the peripheral-target task. (**A**) Schematic of the exogenous mode. A blue vertical bar jumped from the screen center (at the beginning of the trial) to the peripheral target position (upon target onset). (**B**) Schematic of the endogenous mode. For this example, the participant had to direct their gaze to −70° on the left. (**C**) Photograph of GRAIL setup in endogenous mode.

**Figure 2.**
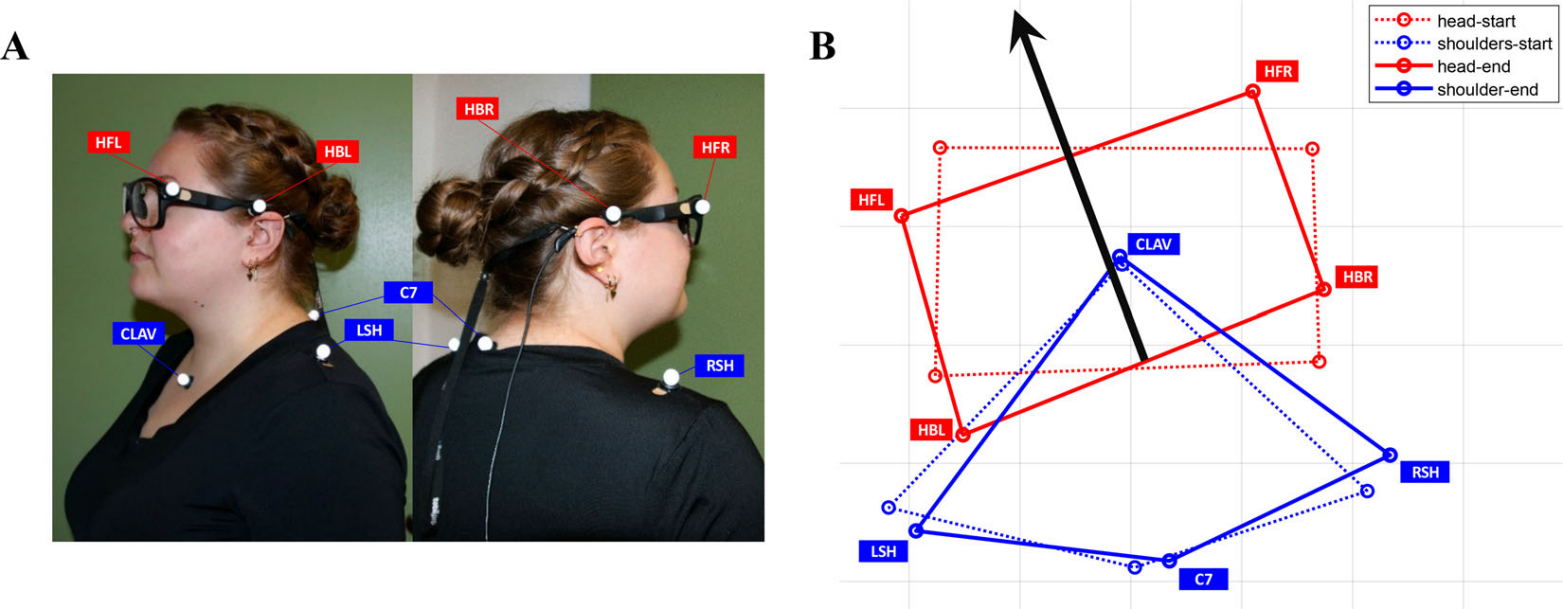
Motion capture markers and model. (**A**) Marker positions as located on the participants (picture shows one of the authors for demonstration). (**B**) Example data from the upper body model (bird's eye view). (**B**, red) Horizontal start and end position of head markers: head front left/right, back left/right. (**B**, blue) Start and end position of torso markers: left/right shoulder, clavicle, C7. (**B**, black) Schematic direction of head vector.

During the peripheral-target task, participants stood in the center of a cylindrical projection screen that is 240° wide and has a radius of 2.5 m (Figure 1). The screen is part of the GRAIL (Gait Realtime Analysis Interactive Lab; Motek Medical, Amsterdam, Netherlands) at TU Chemnitz. The GRAIL system includes five projectors, four of which were used to project the stimuli seamlessly on the screen, the fifth is for projection on the floor. A 10-camera Vicon motion capture system was used for capturing head and torso movements. The treadmill, which is also part of the GRAIL setup, was deactivated throughout the present study. Participants stood in the center of the cylinder encompassed by the panoramic screen. To capture head and torso movements, four retro-reflective markers were placed on the participants’ upper body (right and left shoulder, clavicle, C7) and another four markers were placed on the left and right temple of the Tobii Pro Glasses 3 mobile eye tracker (Tobii AB, Stockholm, Sweden). Together, the eight markers formed an upper body model (Figure 2).

Gaze data were recorded at 100 Hz, the built-in scene camera of the eye-tracking device recorded a video of the scene from the perspective of the participant at 25 Hz with a field of view of 95 degrees horizontally and 63 degrees vertically. Motion capture data were recorded at 250 Hz with Vicon Nexus (Vicon Motion Systems Ltd, Oxford, UK, version 2.8.1); motion capture and screen presentation were controlled and synchronized by D-Flow (version 3.34.3, Geijtenbeek, Steenbrink, Otten, & Even-Zohar, 2011) The Tobii Glasses were synchronized to the system using electronic trigger signals and custom-built hardware.

Motion-capture data were used to compute the location and orientation of head and torso relative to a world-centered reference frame (“head-in-world” [HiW]), eye-tracking data provide data relative to the glasses, which are head-fixed (“eye-in-head” [EiH]). The horizontal EiH component (as defined in head-centered coordinates) and the horizontal HiW component (the projection of the head markers, see below, on the ground plane) were additively combined to obtain the gaze orientation relative to the world (“eye-in-world” [EiW]).

### Stimuli

#### Natural scenes

For the scene-viewing task, we used 84 photos of natural scenes out of the “Uncommon Places” set by Steven Shore (Shore, Schmidt-Wulffen, & Tillman, 2004). Most of these had a surrounding black frame. We removed the frame and cut the images to same resolution (1400 × 1080 px) by centrally cropping them. Stimuli were shown in two sizes: 38.2 × 28.7 dva and 19.1 × 14.4 dva. Each participant saw each scene twice, once per size.

#### Presentation modes of the peripheral-target task

In the peripheral-target task, we used 28 possible target angles along the horizontal axis positioned in steps of 5 dva from −70 to −5 dva and from 5 to 70 dva relative to the center of the panoramic screen (0 dva). The task was carried out in two presentation modes. In the “exogeneous mode” a blue vertical bar was presented either at the screen center or at the peripheral target position (Figure 1A). The bar measured 2 × 8 dva (width × height). In the “endogenous mode” (Figure 1B) participants saw several lines of 1 dva width indicating different eccentricities from the center of the screen: large white lines indicated steps of ten (±10, ±20, ±30, ... ±120 dva), shorter yellow ones indicated steps of five in between (±5, ±15, ±25, ... ±115 dva). Additionally, lines of ±30, ±60, and ±90 dva off the horizontal center were marked with the corresponding value. In the screen center (0 dva), marked by a red line, the target location was instructed in writing. The vertical center of the blue bars in the exogeneous mode and the numbers in the endogenous mode were adjusted individually to a height matching the eye level of the respective participant.

### Procedure

#### Scene-viewing task

Participants were instructed that they will see photos of real-world environments. Their task was to look carefully at the images while they were allowed to move their eyes naturally (in German: “Ihre Aufgabe ist es, die Bilder genau anzuschauen, dabei dürfen Sie Ihre Augen natürlich bewegen.” [You have the task to look at the images carefully, you may move your eyes naturally]). A 13-point-calibration and validation procedure was applied at the beginning of each block. Each trial started with a central fixation cross, which participants had to fixate (i.e., stay within a radius of 1 dva) for at least 300 ms. If the fixation did not fall within this radius for five seconds, the eye tracker was recalibrated. Across all data, the median presentation duration of the fixation cross was 585 ms. After successful fixation, participants viewed a natural scene for five seconds, before the next trial started automatically. In total, there were two blocks. In each block, all 84 images were shown, 42 images per size. Every image was used exactly once per block. For each of the scenes, the size presented in the first block was balanced across participants (the other size being presented in the second block). Within each block the order of images was randomized independently for each participant.

#### Peripheral-target task

First, markers were applied (Figure 2A), motion tracking cameras and Tobii Glasses were calibrated and the calibration validated. During experimental blocks, participants stood upright in the center of the cylinder formed by the panoramic screen setup, which was marked by a circle projected to the floor. At the beginning of each trial, participants had to orient their gaze, head, and torso to the starting direction straight ahead (0 dva).

By pressing a button on a hand-held device, they confirmed that they reached the starting pose and simultaneously triggered the gaze target on the screen. In the exogenous mode, the blue vertical bar was removed from the screen center and reappeared at the peripheral target position upon button press. Participants’ task was to direct their gaze in a natural way to the vertical bar at target position (in German: “Blick in natürlicher Art und Weise zur Position des auftauchenden Balkens ausrichten.” [Direct gaze in a natural way to the location of the appearing bar.]). In the endogenous mode, a number appeared centered at the vertical line at 0 dva indicating the target position and participants’ task was to direct their gaze to the instructed target position (“Blick in natürlicher Art und Weise zur angezeigten Position ausrichten.” [Direct gaze in a natural way to the indicated location.]).

In both modes, the successfully aligned gaze was confirmed by button press and the next trial began. That is, in exogenous mode, the peripheral vertical bar jumped back to the screen center and in endogenous mode, the number indicating the previous target position was replaced by 0.

Each target position was presented once per block. In total, there were 10 blocks so that each target position was presented 10 times per participant (five times per presentation mode). The blocks of each presentation mode were presented alternatingly, and the starting condition was balanced across participants.

### Data extraction, processing, and variables

In the scene-viewing data, fixations, blinks, and saccades were detected by using Eyelink's built-in functions with saccade thresholds of 35°/s for velocity, and 9500°/s^2^ for acceleration. Blinks were removed from the data, which excludes 1.89% of raw eye positions and 9.04% of detected saccades (Eyelink‘s software detects blinks as part of presumed saccade events, which are in fact most often due to the lid closure and opening). On basis of the blink-free data set, we excluded all fixations that fell outside an image; this pertained to 0.29% of fixations in large images and 0.40% of fixations in small images. Saccades were only included if they started and ended within the image, leading to an exclusion of 0.46% (large images) and 0.72% (small images) of saccades.

**Figure 3.**
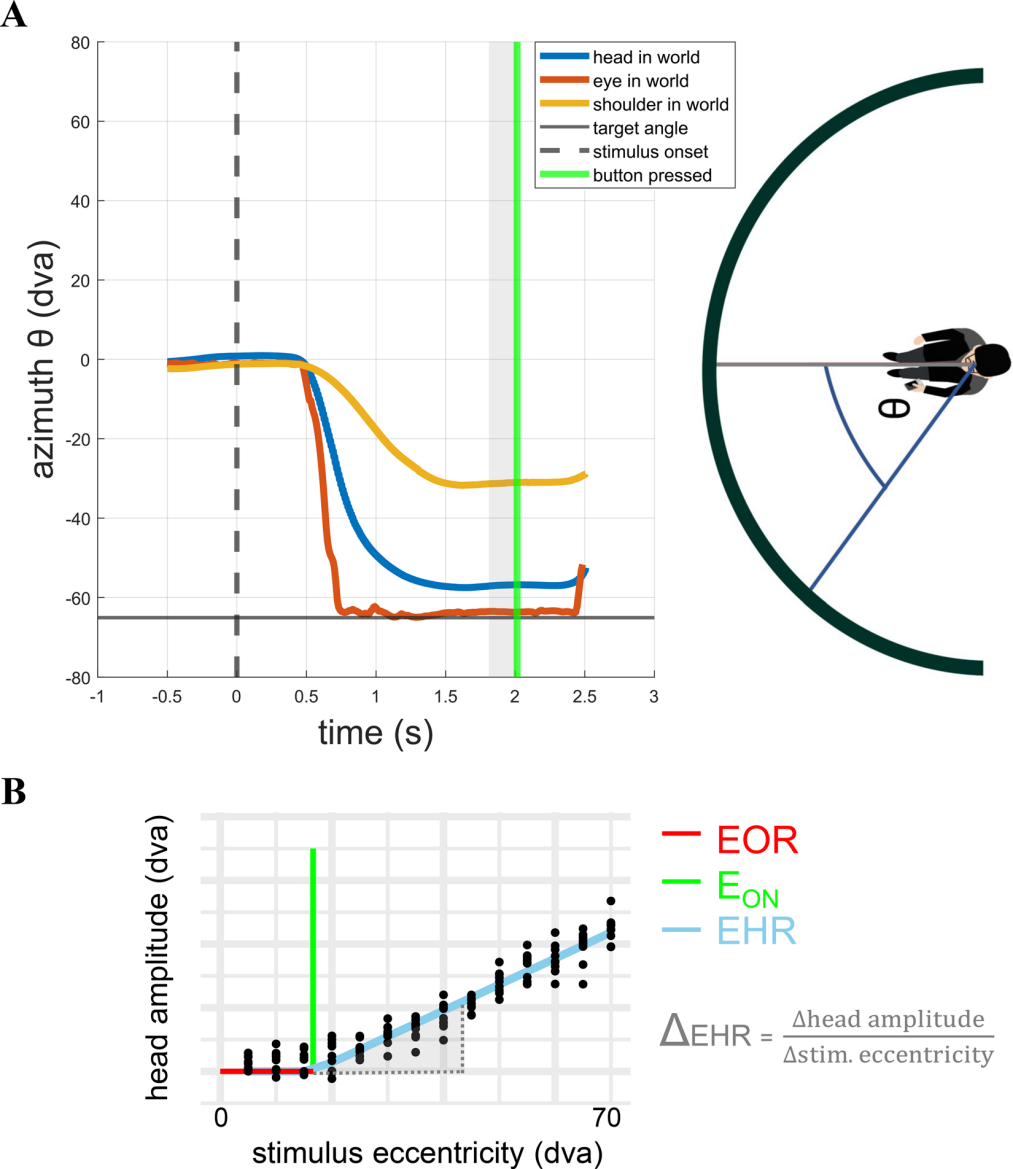
Extraction of the azimuthal effector orientations. (**A**, right): Schematic illustration of computed azimuthal angles θ between horizontal projection-screen-center-vector and head-direction vector. (**A**, left) Corresponding example trial data including estimated orientation of eye- and shoulder vector. For each trial, orientations were extracted by averaging a 200-ms-window (light gray) before the button press of the participant that confirmed the fixation of the target position (green). (**B**) Example data of one participant for illustration of eye-only-range (EOR), head-onset threshold (E_ON_), and eye-head-range (EHR). The slope (Δ_EHR_) corresponds to the head contribution of gaze-target-alignment inside the EHR. Data was collapsed for right/left side (±) by taking the absolute of head amplitude and target angle. Negative head amplitudes connotate final head positions pointing away from target direction (i.e., to the opposite hemisphere).

For the peripheral-target task, mobile eye-tracking data was imported to MATLAB by using the GlassesViewer Toolbox (Niehorster, Hessels, & Benjamins, 2020). We interpolated data gaps smaller than 100 ms with a cubic spline method (Hunt, Blackmore, Mills, & Dicks, 2022) using the MATLAB built-in function “fillmissing” (method = “spline,” “EndValues” = “none,” “MaxGap” = 100 ms). For saccade detection we used a velocity threshold of 35°/s. From motion tracking, we gathered three-dimensional position data. We computed the horizontal head orientation from the four head markers (Figure 2, red) by averaging the positions of frontal (HFL, HFR) and back markers (HBL, HBR), projecting the position onto a plane parallel to the ground, and calculating the azimuthal (horizontal) angle between this projection of the head vector (HiW) and a vector pointing straight to the horizontal center of the projection screen (Figure 3A, right). We excluded trials in which the final EiW angle differed more than 5 dva from the target angle. Additionally, we excluded trials in which the EiW angle was initially (average over the time window from 0 to 1s after trial onset) directed to the hemifield opposite to the target side. Together, this led to the exclusion of 4.81% of all trials. This number of excluded trials was not equally distributed across presentation modes and target positions. For the endogenous mode we found generally more initial direction errors (8.2% of all endogenous trials) than for the exogenous mode (1.4% of all exogenous trials). The mean deviation of EiW from the horizontal center (0 dva) in endogenous mode scattered inside the inner −20 to 20 dva area. Compared to exogenous mode (scattering inside the inner −40 to 40 dva area), this was a smaller deviation from the center, which could be attributed to the fact that participants first needed to process the centrally written target angle before moving forward to their gaze destination. Across target angles, we found more initial direction errors at the outer angles (60 to 70 dva on both sides) compared to the central ones. However, as the number of excluded trials was in general low, we consider it exceedingly unlikely that a bias resulting from these differences affects our results.

For general data pre-processing MATLAB (version R2022b) was used. All subsequent analysis steps were performed in R (Posit team, 2023).

#### Central bias in scene viewing

To estimate individual central biases from the free scene-viewing data, we computed the Euclidean distance of each fixation to the image center and took the median for each participant and image size. Fixations were included, if they started after image onset and before image offset; that is, the initial central fixation that started prior to image onset was not included.

#### Head-movement propensity

 From the three-dimensional position data of the peripheral-target task, we computed the azimuthal (horizontal) angle between the horizontal screen center and the HiW vector (Figure 3A). For each trial, we used the median angle of the 200-ms-time-window before the button press that confirmed gaze alignment to the target to obtain the final orientation of the head.

When plotting head amplitudes as a function of gaze-target eccentricity, one usually expects a linear graph with two breakpoints, i.e. points from where on the linear relation of these variables changes (Oommen et al., 2004; Proudlock & Gottlob, 2007; Stahl, 1999; Thumser, Oommen, Kofman, & Stahl, 2008). The central part of this graph (around 0 dva target angle) corresponds to the Eye-Only-Range (EOR), where the target gaze position is mainly reached by using eye movements and nearly no head movements are used. To reach target positions outside the EOR, humans use a combination of eye and head movements. These intervals to the left and right of the EOR are called eye-head-range (EHR).

In our analysis we collapsed the data of the left and right side of the center (0 dva) by taking the absolute value of the head amplitude and of the target angle. This means we only get a single EHR interval instead of two and only a single breakpoint of the graph. As measures of individual head-movement propensity, we computed two-piece piecewise linear fits for each participant and presentation mode as follows: the initial part of the function is assumed to be constant at 0 dva (no head usage) up to a head-onset threshold (E_ON_). Target eccentricities smaller than E_ON_ correspond to the EOR. Beyond E_ON_ (i.e., inside the EHR), the function increases linearly with a constant slope (Δ_EHR_) that quantifies the contribution of head movements to gaze-target alignment in the EHR (also known as “head gain”, Figure 3B). The function with the two parameters E_ON_ and Δ_EHR_ is fitted using the “segmented” package in R (Muggeo, 2008). This function segments the data along the x-axis (in our case the target-eccentricity), applies a linear model to each segment and identifies the value of x where the split works best, which is the estimated breakpoint E_ON_.

We ran a control analysis computing the slope for the combined eye and head movements (EiW) analogously to the slope for the head contribution (Δ_EHR_). Unless there were biases in the participants’ overall gaze shifts to the targets, this should provide a slope indistinguishable from one. On average we got a slope close to 1 (mean: 1.005, standard deviation: 0.021, range: 0.946 to 1.037), which confirmed that participants complied with instruction and that the small deviation resulting from the distinct coordinate systems for EiH (referenced to the head) and HiW (referenced to the world) are negligible.

#### Comparing scene-viewing data to head-movement propensity

To quantify a possible (linear) relation between central bias and each of the two measures of head-movement propensity, we computed Pearson correlations. Similarly, we computed Pearson correlations of other variables obtained from the scene-viewing data (e.g., fixation duration) with the head-movement-propensity measures. In addition to central bias, we analyzed the dependence of fixation duration and saccade amplitude on the two head-movement propensity measures, using the same analysis approaches. Since central bias is known to change over viewing time (e.g. Rothkegel et al., 2017; Tatler, 2007), even if the initial fixation is non-central (Backhaus & Engbert, 2024), we also analyzed the temporal evolution of this measure. To this end, we either used 500-ms bins or the ordinal fixation number as time base.

**Figure 4.**
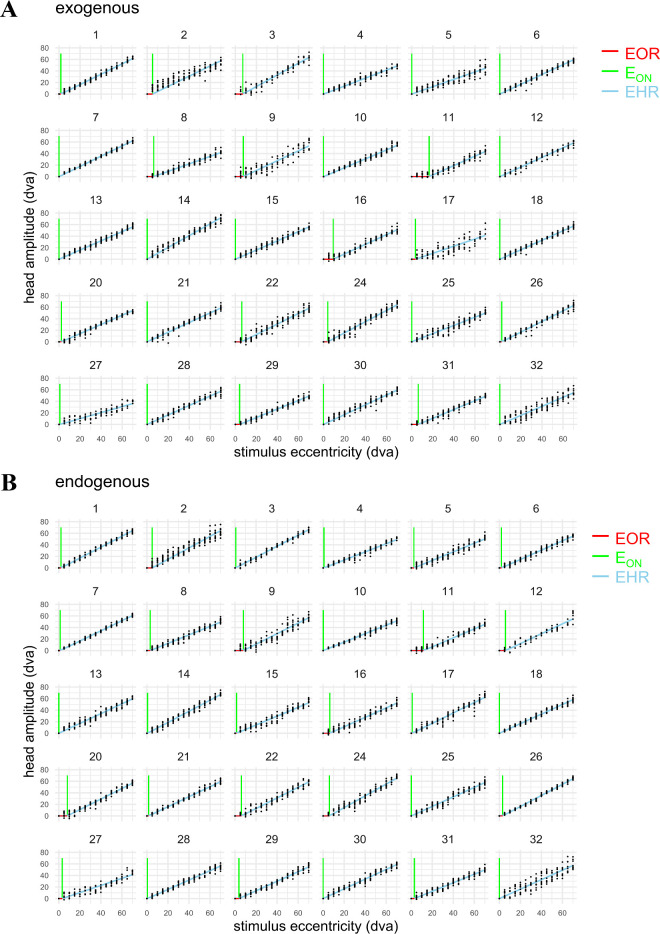
Two-piece piecewise linear fits for the estimation of individual head propensity in exogenous (**A**) and endogenous (**B**) condition. In both presentation modes, most participants started contributing their head for the final gaze-target-alignment within the smallest target eccentricity tested (5 dva). Participant IDs 19 and 23 were excluded for lack of data after applying trial exclusion criteria prior to this analysis, IDs 11 and 27 were excluded from some analyses because of extreme values in at least one of these measures (see text for details).

All analyses described so far average central bias over the whole image ensemble and – with exception of the time-binned analysis – across all fixations of an individual. As an additional analysis, we computed linear mixed-effects models (LMM) using each fixation as an observation and either the normalized distance to the center or the logarithm of the fixation duration as dependent variable. In both cases, we used both head-movement propensity measures (E_ON,_ Δ_EHR_) as fixed effects as well as image and participant as random factors (Table 1). Besides computing LMMs for the whole viewing duration (0–5000ms), we also used a time-binned analysis using – following Backhaus & Engbert (2024) – time bins of 0–400ms, 400–800ms, 800–1200ms, and 1200–end of viewing duration (5000 ms). Unless otherwise stated, two participants (randomly assigned ID 11 and 27 in Figure 4) were excluded from the LMM analysis, because at least one of their head-movement propensity measures deviated so substantially from all others that they would bias the model's results substantially (see section in Results “Exclusion of data for further statistical analysis”). LMM-analysis was performed in R (Posit team, 2023) and the lme4 package (Bates, Mächler, Bolker, & Walker, 2015). Model estimation was performed via maximum likelihood and the Bobyqa optimizer. The *p* values were calculated with the lmerTest package (Kuznetsova, Brockhoff, & Christensen, 2017).

**Table 1.** Linear mixed-effects model structure in Wilkinson notation. *Notes*: Random effects: Subj = participant; Img = image; Size = image size [not included for central bias, because we use the normalized distance]. Fixed effects: E_ON_ = head onset threshold; Δ_EHR_ = head contribution; InitSacLat = initial saccade latency; log(fixT) = logarithm of time to trial-onset; log(sampleID) = logarithm of sample number (i.e., time since experiment onset); the latter two predictors were included as covariates to control the change of central bias over trial time and time course of the experiment, respectively (cf., Backhaus & Engbert, 2024); all continuous variables were normalized to zero mean (centered) and unit standard-deviation (z-scaled).

Dependent variable	Fixed effects part	Random effects part
0–5000 ms		
Normalized distance to center ∼	1 + E_ON_ + Δ_EHR_ + log (fixT) + log (sampleID)	+ (1|Img) + (1|Subj)
log (fixation duration) ∼	1 + E_ON_ + Δ_EHR_ + log (fixT)	+ (1|Img) + (1|Subj) + (1|Size)
Time-binned		
Normalized distance to center ∼	1 + E_ON_ + Δ_EHR_ + log (sampleID)	+ (1|Img) + (1|Subj)
Log (fixation duration) ∼	1 + E_ON_ + Δ_EHR_	+ (1|Img) + (1|Subj) + (1|Size)
Normalized distance to center (second fixation) ∼	1 + E_ON_ + Δ_EHR_ + InitSacLatency	+ (1|Img) + (1|Subj)

## Results

### Head-movement propensity from peripheral-target task

Thirty participants were included in the analysis of the peripheral-target task. We fitted their individual head-onset threshold (E_ON_) and head contribution in the EHR (Δ_EHR_) separately for the two presentation modes (Figure 4A for the individual exogeneous, Figure 4B for individual endogenous data and fits).

**Figure 5.**
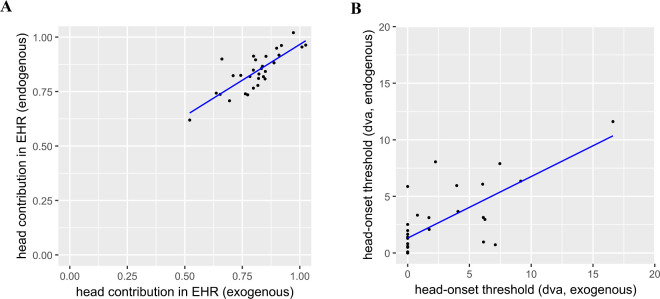
Correlation of head contribution in the EHR (Δ_EHR_, A) and head-onset-threshold (E_ON_, B) across presentation modes.

For Δ_EHR_, we found a strong positive correlation between presentation modes (*r*(28) = 0.80, *p* < 0.001, Figure 5A). Although their means differed (*t*(29) = −2.65, *p* = 0.01), the strong correlation justifies combining the results for the two presentation modes into an average value for each individual. We used this average for comparison with the central-bias data from the scene-viewing task (considering exogeneous and endogenous mode separately yields the same conclusions).

As is evident from the individual data (Figure 4), most participants started using their heads for final gaze-target-alignment within the smallest target eccentricity tested (5 dva). Hence, the estimation of the head-onset threshold (E_ON_, Figure 5B) is likely to be less robust than the head-contribution estimation in the EHR (Δ_EHR_, Figure 5A). We found a moderate positive correlation between the presentation modes (*r*(28) = 0.72, *p* < 0.001), which persisted when the outlier (Figure 5B, upper right; participant ID 11; details on exclusion see below) was removed (*r*(27) = 0.56, *p* = 0.002) for E_ON_. Because we have no evidence that E_ON_ differs between the two presentations modes (outlier included: *t*(29) = –0.21, *p* = 0.83; outlier excluded: *t*(28) = −0.58, *p* = 0.57), we averaged the two E_ON_ measures of each participant for further analysis.

In our analysis, we mainly focus on spatial aspects of eye-head-coordination. However, there is also a temporal component which is known to differ between exogenous and endogenous attention shifts (Doshi & Trivedi, 2012). Saccadic eye movements started later for endogenous (mean latency: 625 ms ± 26.0 ms) than for exogenous cues (253 ms ± 12.4 ms; *t*(29) = −17.75, *p* < 0.001), but in contrast to Doshi & Trivedi (2012) we did not see systematic differences in head movements preceding the saccade. Neither the (absolute) deviation of the head orientation from the direction straight ahead (2.07 dva and 2.16 dva respectively, *t*(29) = −0.39, *p* = 0.70) nor the head velocity (0.023°/s and 0.018°/s, *t*(29) = 1.79, *p* = 0.08) differed between our exogenous and endogenous presentation mode in the 100-ms interval before saccade start.

### Central bias from scene-viewing task

**Figure 6.**
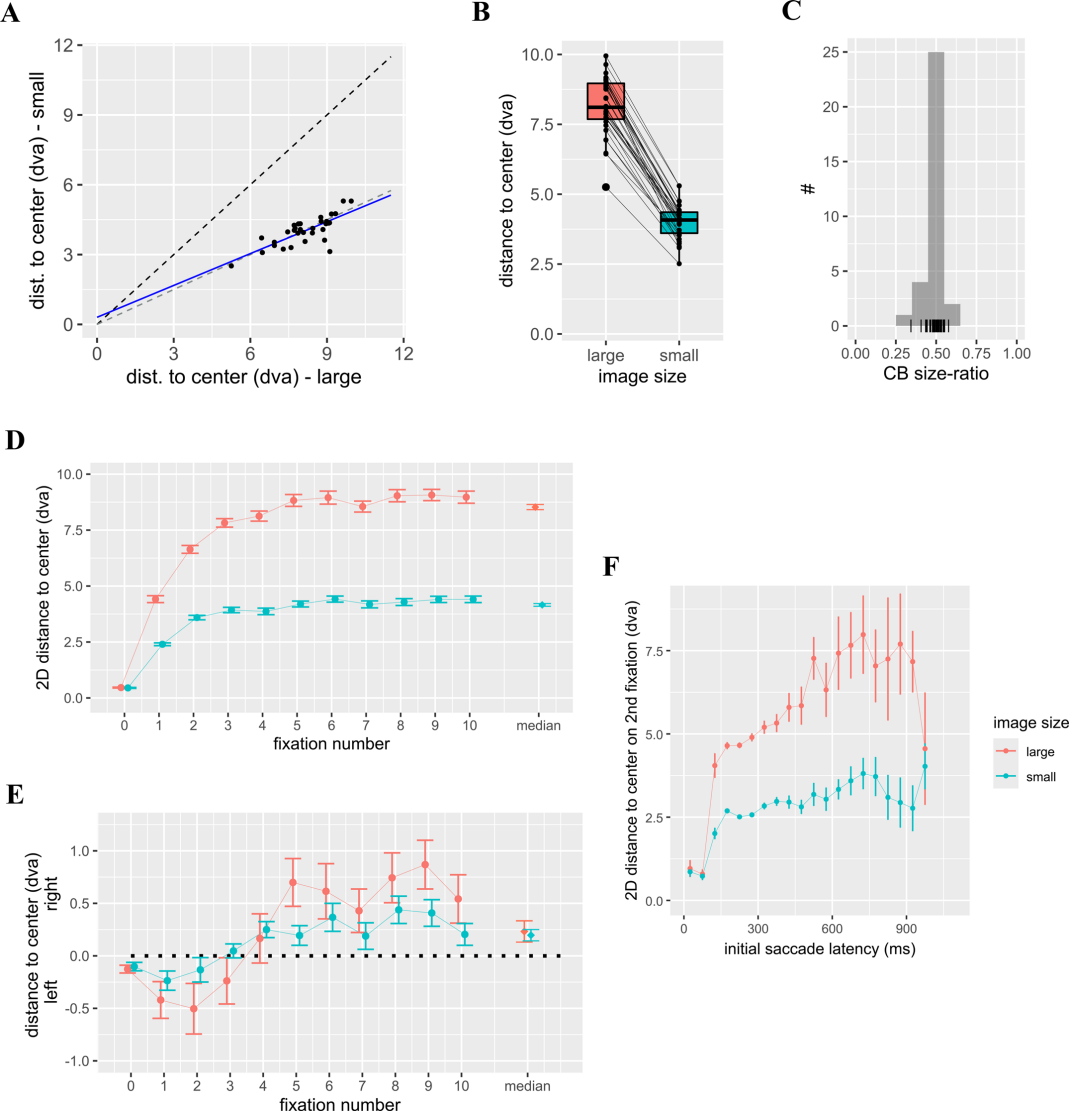
Central bias (CB) across the two image sizes. Smaller distances to the screen center indicate a stronger central bias. (**A**) Individual central bias. Dashed black line: slope = 1, dashed gray line: slope = 0.5, blue line linear fit: CB_small images_ = 0.31 dva + 0.46 × CB_large images_. (**B**) Size difference in central bias. (**C**) Distribution of individual ratios CB_small images_/CB_large images_. Binwidth = 0.1. Mean ratio: 0.494. Lines indicate participant-specific ratios. (**D**) Euclidian distance to center and (**E**) horizontal distance to center across fixation numbers. Both medians ± SEM. (**F**) Central bias (means ± SEM) of the fixation after the initial saccade as a function of the saccade's latency.

Participants saw 84 images in two different sizes each. We quantified an individual's central bias (CB) for each image size by computing the median distance of their fixations from the image center. Between the two image sizes we found a strong positive correlation (*r*(30) = 0.77, *p* < 0.001, Figure 6A) in this measure of central bias. If central bias is predominantly stimulus-driven, we expect it to scale with image size. That is, the central bias should be half as large for small images compared to large ones. There indeed was a significant difference in distances between the two sizes (*t*(31) = 33.91, *p* < 0.001; Figure 6B) with a mean ratio between the two conditions (CB_small images_/CB_large images_, Figure 6C) of 0.494. This value was statistically undistinguishable from 0.5 (*t*(31) = −0.67, *p* = 0.51, Figure 6C). This implies that the extent of the central bias scales with image size, at least for the image sizes tested.

Central bias develops over time, but the ratio between the two image sizes stays more or less constant at 0.5 (Figure 6D). The definition of central bias implicitly assumes that the bias develops symmetrically around the center; however, it is well-known that there is a slight tendency to the left in the beginning of scene viewing, which rebounds to the right in later phases (“pseudoneglect” [Nuthmann & Matthias, 2014; Ossandón, Onat, & König, 2014], for a similar left-ward bias see Engmann et al., 2009). This is also observed for the present data (Figure 6E), but negligible in size compared to the central bias as such. Interestingly, the image-size dependence seems to be maintained for the tiny deviations from the center. The “central” bias shows over fixation number, with an initial bias to the left (pseudoneglect) and a later rebound to the right (Figure 6E). Moreover, the central bias of the second fixation is enlarged (i.e., its distance to the center reduced) if the latency of the preceding saccade is low (Figure 6F), consistent with previous results (Rothkegel et al., 2017; see also below). In general, central bias effects scale with image size.

 The consistency across sizes allows us to obtain a single measure of central bias in each participant. To this end we first normalized each distance to image center by dividing it by its corresponding stimulus diagonal and averaged it across the two sizes. This results in a number between 0 and 0.5, where 0 would correspond to looking at the center of the image, and 0.5 at its corners; a stronger central bias is therefore implied by a *smaller* number.

### Exclusion of data for further statistical analysis

In the following, we will consider relations between the measures of head-movement propensity (E_ON_, Δ_EHR_) on the one hand and central bias, as well as other gaze-parameters on the other hand. The distribution of E_ON_ has a median of 1.58, and 29/30 datapoints fall within the interval from 0 to 7.75, while one single datapoint (participant ID 11) nearly doubles this upper bound at a value of 14.1 (cf. Figure 5B). Similarly, for Δ_EHR_ the median is 0.83, the range of all but one datapoint reaches from 0.69 to 1.00 and the remaining datapoint (participant ID 27) has a value of 0.57, which is outside two times the interquartile range ([0.78, 0.88]). To avoid our data being biased by these extreme datapoints, we report analyses pertaining to the respective head-movement propensity measure with these outliers excluded (see the respective analysis for details), but also for the full *N* = 30 sample for transparency.

### Relation of head-movement propensity and central bias

**Figure 7.**
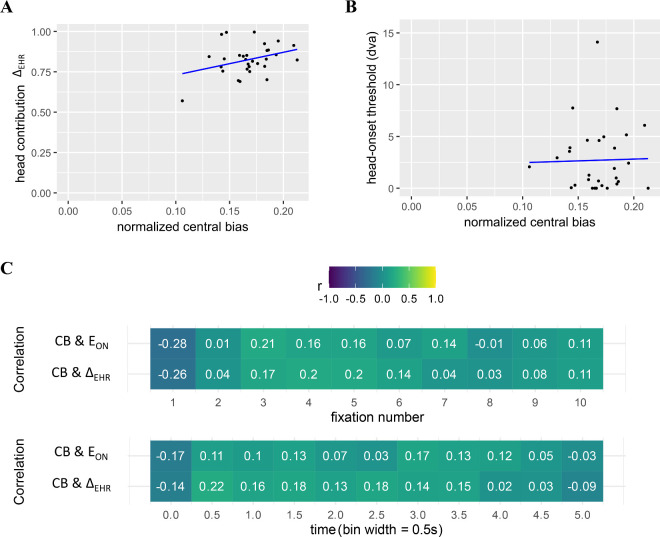
Correlation of individual central bias with slope Δ_EHR_ (**A**) and threshold E_ON_ (**B**). (**C**) Correlation coefficients for correlations of fixation-number- and time -specific central bias with E_ON_ (top rows) and Δ_EHR_ (bottom rows) for *N* = 28 (ID 11 and 27 removed). None of the correlations was statistically significant (all *p* > 0.05).

The core hypothesis of this study was a presumed dependence of an individual's head-movement propensity to their central bias. We could estimate both measures robustly, and both measures show inter-individual variability and intra-individual consistency that allows us to find robust correlations between different measurement methods (exogenous/endogenous and large/small image, respectively). Nonetheless, the correlation of normalized central bias with either measure of head-movement propensity was indistinguishable from 0 (correlation with Δ_EHR_: *r*(28) = 0.34, *p* = 0.07; Figure 7A; correlation with E_ON_: *r*(28) = 0.02, *p* = 0.90; Figure 7B). The trend to significance for Δ_EHR_ is entirely driven by the outlier (visible to the lower left of Figure 7A), and the correlation reduces to *r*(27) = 0.11, *p* = 0.57 if this outlier (ID 27) is excluded and to *r*(26) = 0.11, *p* = 0.59, if both outliers (ID 11 and ID 27) are excluded. For E_ON_, removing the outlier (ID 11, visible on the top of the plot) yields a correlation of *r*(27) = 0.04, *p* = 0.85, removing both (ID 11 and ID 27) yields *r*(26) = 0.03, *p* = 0.87. Hence, the correlation analysis shows no robust relation between head-movement propensity and central bias, and the only statistical trend is likely attributable to a single outlier.

**Figure 8.**
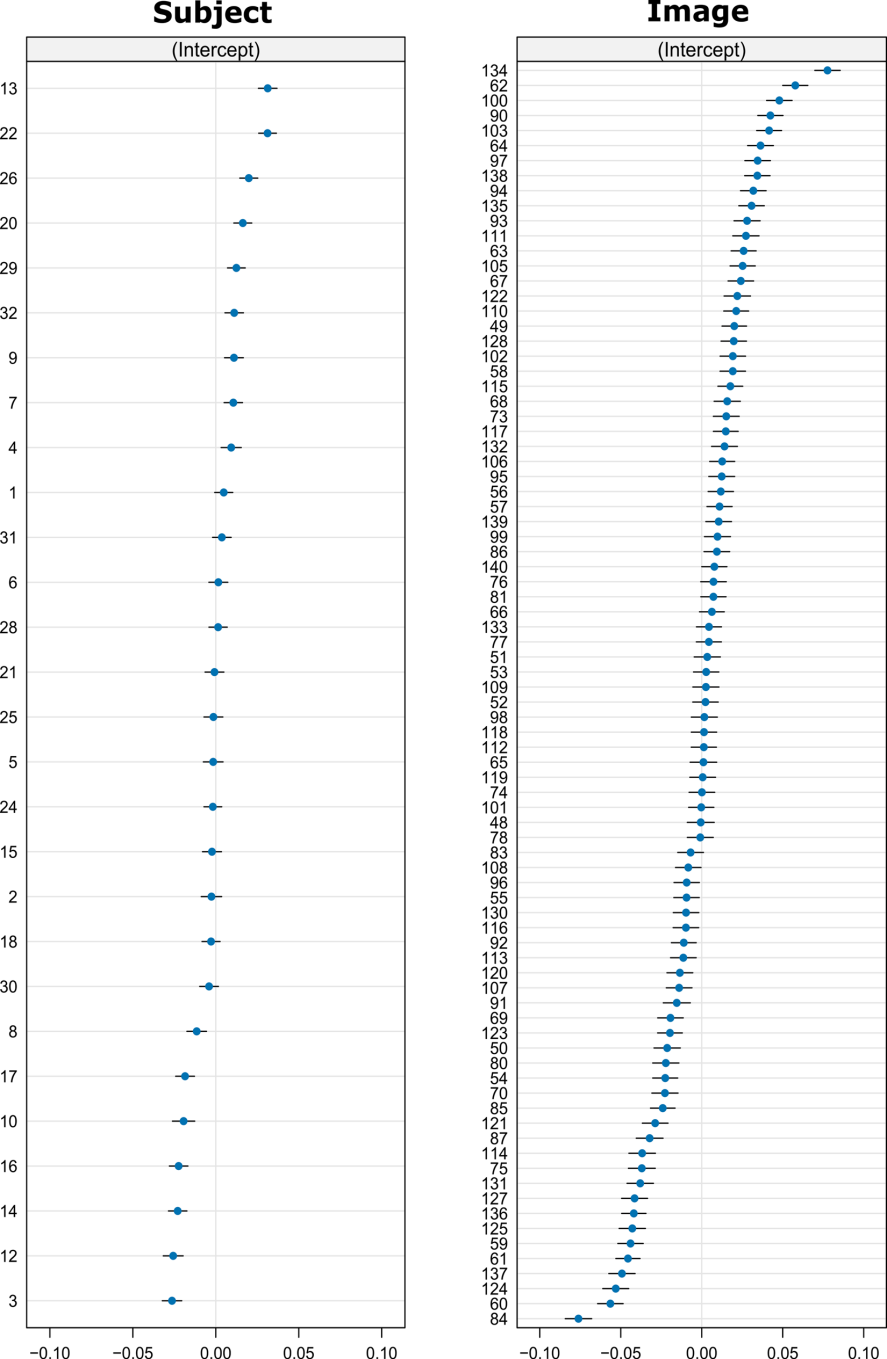
Random effect of subject and image for normalized Central Bias (0-5000 ms), deviation from mean*.* Left: participant (IDs correspond to Figure 4); right: image (IDs match the original database and are not consecutively numbered).

Since central bias is known to change across viewing time (Tatler, 2007), we additionally computed individual, fixation-number-specific and time-binned central biases and correlated them with individual head-propensity measures. When excluding the two individuals with extreme data in head-movement propensity (IDs 11 and 27) from analysis, we find the correlation to be close to zero in all time bins (all |*r*|<0.28, all *p* > 0.14; Figure 7C). When including these two participants, correlations get—as expected—somewhat larger, but all remain below 0.41, and none is significantly different from zero after correction for multiple comparisons, even when a liberal correction is used (at 5% expected false-discovery rate according to Benjamini & Hochberg, 1995; all uncorrected *p* > 0.023).

To consider fixation-level data of central bias instead of averaged data, we in addition computed LMMs (see Table 1 for LMM structure). Normalized distance to image center was used as measure of central bias and as dependent variable in the LMMs. The individual head-propensity measures (E_ON_ and Δ_EHR_, averaged over presentation modes) were used as fixed effects to predict normalized distance to center. To predict log fixation duration, the time relative to trial-onset and the sample number were incorporated as additional co-variates to capture a potential change of central bias over trial time and the course of the experiment, respectively (cf., Backhaus & Engbert, 2024).

Across the whole viewing time (0-5000ms) as well as across all time-bins (0–400 ms, 400–800 ms, 800–1200 ms, 1200–5000 ms), we found no significant effect of head-onset threshold (E_ON_) and head contribution (Δ_EHR_) on normalized distance to center (Table 2, left). When including the two outliers, some LMMs show (marginally) significant predictions by head-movement propensity for some time bins (Table 2, right), which is consistent with the same outliers driving the trend to significance in the correlation (Figure 7A). However, given the extreme difference in head-movement propensity between the outliers and the—in itself highly variable—remainder of the sample, this is likely to be a brittle and spurious effect.

**Table 2.** LMM—normalized central bias. *Notes*: Fixed effects parts, |t| > 2 are interpreted as significant, left part of table corresponds to LMMs without outliers (*N* = 28), right part to LMMs including outliers (*N* = 30). Values less than 0.005 are denoted at “0.00” except for *p* values. Random effects for the first model (0–5000 ms, *N* = 28) are shown in Figure 8.

	*N* = 28 (ID 11 and 27 excluded)				*N* = 30			
Predictors	Estimate *β*	*SE*	*t*	*p*	Estimate *β*	*SE*	*t*	*p*
Normalized Distance to Center 0–5000 ms								
Intercept	0.18	0.00	**41.98**	<0.001	0.18	0.00	**41.28**	<0.001
E_ON_	0.00	0.00	0.45	0.652	0.00	0.00	−0.01	0.989
Δ_EHR_	0.00	0.00	0.62	0.534	0.01	0.00	**2.12**	0.034
Normalized Distance to Center 0–400 ms								
Intercept	0.11	0.00	**28.58**	<0.001	0.11	0.00	**28.50**	<0.001
E_ON_	0.00	0.00	−0.84	0.400	0.00	0.00	−0.33	0.741
Δ_EHR_	0.00	0.00	−0.82	0.413	0.00	0.00	−0.23	0.815
Normalized Distance to Center 400–800 ms								
Intercept	0.16	0.01	**28.59**	<0.001	0.16	0.01	**28.78**	<0.001
E_ON_	0.00	0.00	0.45	0.655	0.00	0.00	0.28	0.776
Δ_EHR_	0.00	0.00	1.07	0.286	0.01	0.00	**2.12**	0.034
Normalized Distance to Center 800–1200 ms								
Intercept	0.17	0.01	**30.92**	<0.001	0.17	0.01	**31.62**	<0.001
E_ON_	0.00	0.00	0.26	0.796	0.00	0.00	0.23	0.822
Δ_EHR_	0.00	0.00	0.87	0.385	0.01	0.00	1.85	0.624
Normalized Distance to Center 1200–5000 ms								
Intercept	0.19	0.01	**40.33**	<0.001	0.19	0.00	**39.37**	<0.001
E_ON_	0.00	0.00	0.49	0.625	0.00	0.00	−0.09	0.932
Δ_EHR_	0.00	0.00	0.52	0.606	0.01	0.00	1.98	0.047

**Figure 9.**
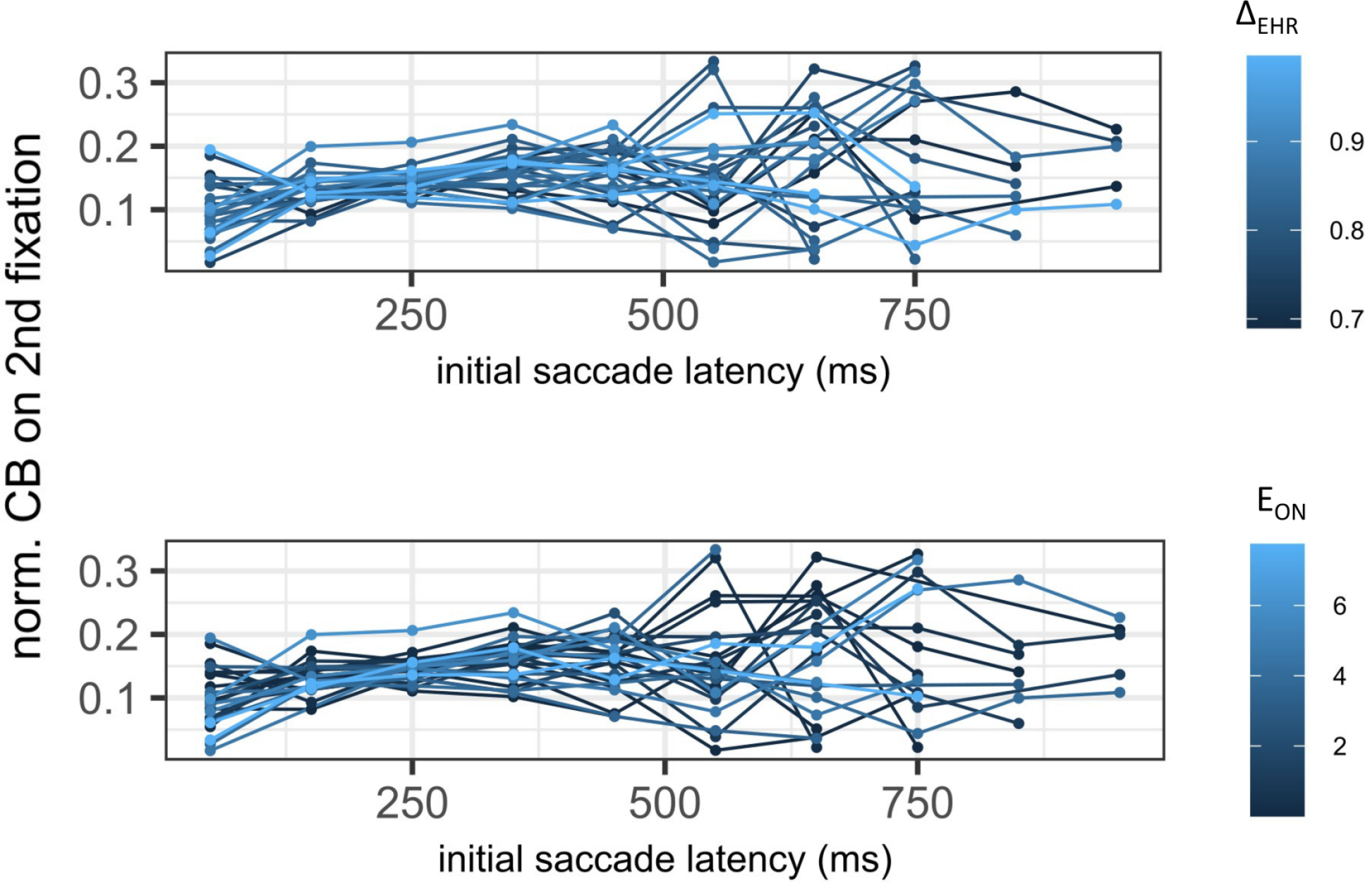
Normalized central bias and its relation to head-propensity measures across initial saccade latencies. Each line corresponds to a unique level of the respective head-movement propensity measure.

Earlier work has shown that the extent of central bias of the first fixation after the initial saccade (i.e., the second fixation on the image) depends on the latency of this saccade (Rothkegel et al., 2017). To check whether we find the same effect in our data, we computed an LMM for the normalized distance of this fixation from the center as dependent variable and latency—in addition to our head-propensity measures—as fixed effect predictors (see Table 1 for model structure). For the full model we included interaction terms of both head-movement propensity measures and initial saccade latency. Because the Likelihood-ratio-test between the full model and a model without both interaction terms indicated no significant difference (and the interaction terms showed no significant effect), we continued with the simpler model without interactions (Table 1). Consistent with Rothkegel et al.’s data, there was a significant effect of initial saccade latency on normalized central bias of the second fixation (Table 3; Figure 9). The two head-propensity measures, however, showed no effect, which is in line with our other analyses so far.

**Table 3.** LMM—normalized central bias of second fixation. *Notes*: Fixed effects parts, |t| > 2 are interpreted as significant, left part of table corresponds to LMMs without outliers (*N* = 28), right part to LMMs including outliers (*N* = 30). Values less than 0.005 are denoted at “0.00” except for *p* values.

	*N* = 28				*N* = 30			
Predictors	Estimate *β*	*SE*	*t*	*p*	Estimate *β*	*SE*	*t*	*p*
Normalized distance to center of second fixation (0–1000 ms)								
Intercept	0.15	0.01	**29.54**	<0.001	0.15	0.01	**29.47**	<0.001
E_ON_	0.00	0.00	0.03	0.980	0.00	0.00	−0.32	0.749
Δ_EHR_	0.00	0.00	0.39	0.696	0.00	0.00	1.53	0.126
Initial Saccade Latency	0.00	0.00	**5.89**	<0.001	0.01	0.00	**5.53**	<0.001

### Effects of image size on other gaze parameters and their relation to head propensity

**Figure 10.**
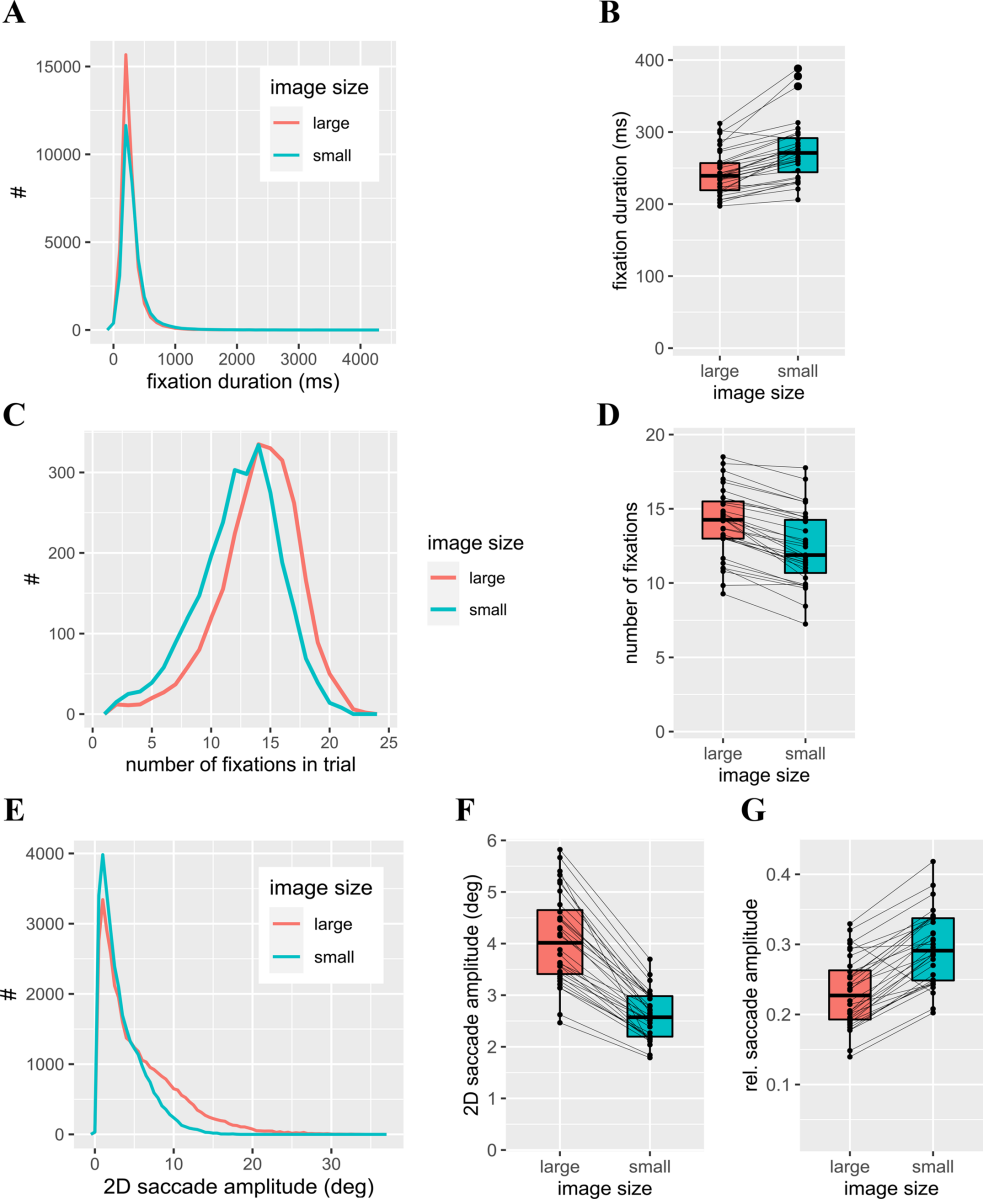
Other gaze parameters across the two image sizes. (**A**) Distribution of fixation duration. Bin width: 100 ms. (**B**) Median individual fixation duration. (**C**) Distribution of the number of fixations per trial. Bin width: 1. (**D**) Mean individual number of fixations per trial. (**E**) Distribution of absolute saccade amplitude. Bin width: 0.5 dva. (**F**) Median individual saccade amplitude. (**G**) Median individual saccade amplitude relative to corresponding image diagonal.

Although the study was designed to assess a potential dependence between head-movement propensity and central bias as defined above, we analyzed some additional variables of scene viewing exploratively, whose relation to scene size might be of interest beyond the original research question. Fixation duration (Figures 10A, B) depended on image size with longer durations for smaller images (*t*(31) = −8.21, *p* < 0.001), but there was a strong correlation between fixation durations for the two image sizes across participants (*r*(30) = 0.89, *p* < 0.001), highlighting a high intra-individual consistency.

Number of fixations (Figures 10C, D) also depended on image size. On average, participants performed more fixations on larger images (*t*(31) = 11.50, *p* < 0.001) and showed high intra-individual consistency (*r*(30) = 0.94, *p* < 0.001) between image sizes.

Saccade amplitudes (Figures 10E through 10G) showed a general tendency to smaller saccades which was more pronounced in small images. Median saccade amplitudes differed significantly between image sizes (*t*(31) = 13.81, *p* < 0.001). For relative saccade amplitudes (saccade amplitudes divided by the corresponding image diagonal; Figure 10G), the effect of image size changed its direction: for large images we found significantly larger relative saccade amplitudes (*t*(31) = −10.59, *p* < 0.001). Again, there was a strong correlation between relative saccade amplitudes in the two size conditions (*r*(30) = 0.78, *p* < 0.001).

**Figure 11.**
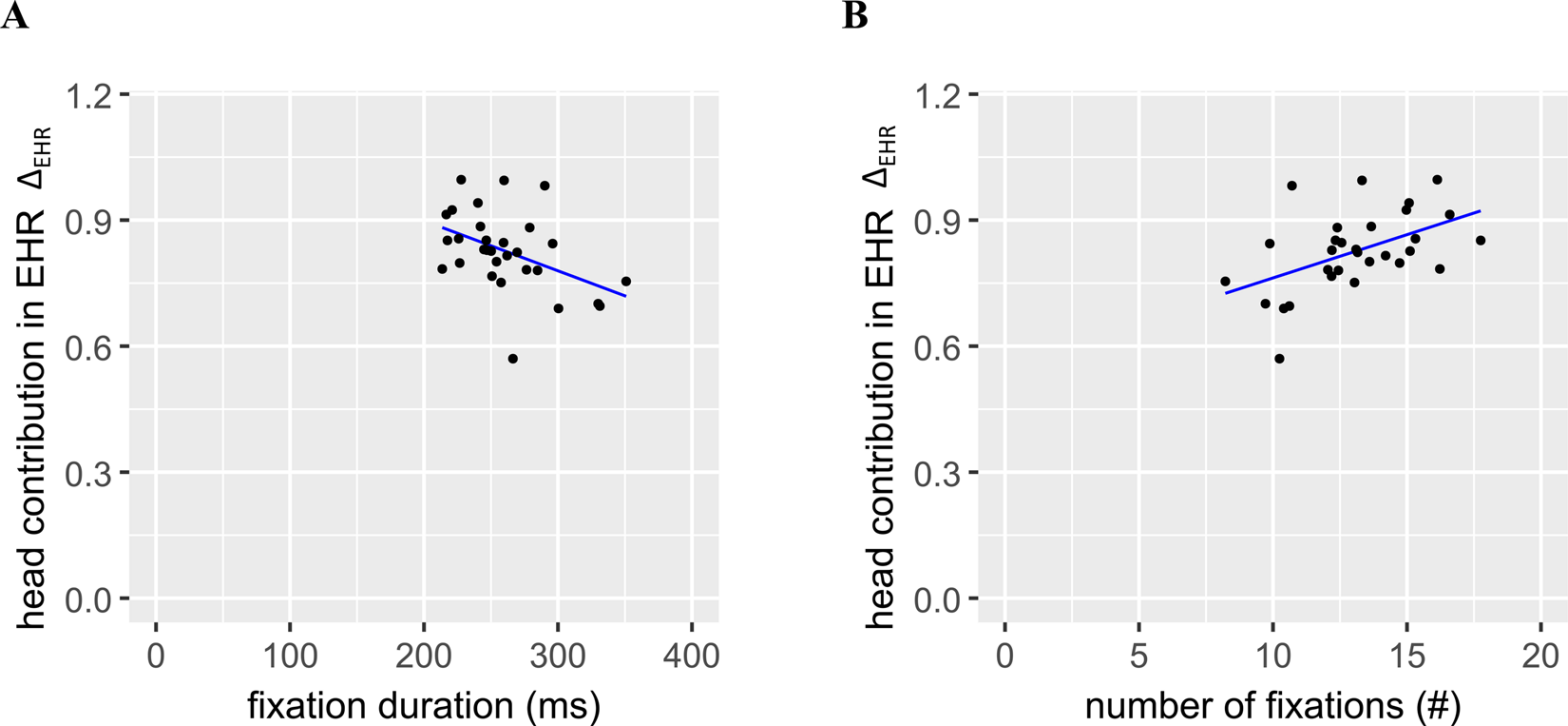
Correlation of EHR-slope (Δ_EHR_) from peripheral-target task with fixation duration (**A**) and number of fixations (**B**) in scene viewing under head-fixed condition.

Besides the effects of image size, we exploratively analyzed the relation of these gaze parameters from scene viewing and head propensity. For E_ON_ there was no significant correlation with fixation duration (with outliers included: *r*(28) = −0.23, *p* = 0.23; with outliers excluded: r(26) = –0.14, *p* = 0.48), number of fixations (*r*(28) = 0.20, *p* = 0.29; *r*(26) = 0.14, *p* = 0.45) and relative saccade amplitude (*r*(28) = −0.05, *p* = 0.77; *r*(26) = 0.12, *p* = 0.53). For Δ_EHR_ the correlation with relative saccade amplitude (*r*(28) = −0.16, *p* = 0.41; *r*(26) = −0.18, *p* = 0.35) was not significant. Somewhat surprisingly, we found a significant moderate correlation between Δ_EHR_ and fixation duration (*r*(28) = −0.44, *p* = 0.01, Figure 11A; *r*(26) = −0.52, *p* = 0.004). Because fixation duration is reciprocally linked to the total number of fixations due to the fixed image presentation duration, Δ_EHR_ was also correlated to the number of fixations (*r*(28) = 0.48, *p* = 0.01, Figure 11B; *r*(26) = 0.45, *p* = 0.02)

Our LMM analysis confirms this finding, we find significant prediction of fixation duration by the head contribution slope (Δ_EHR_), though not by head onset threshold (E_ON_); this still holds when restricting the analysis to the first two seconds (Table 4). In sum, a larger head contribution correlates with shorter fixation durations under head-fixed conditions.

**Table 4.** LMM—fixation durations. *Notes*: Fixed effects parts, |*t*| > 2 are interpreted as significant, left part of table corresponds to LMMs without outliers (*N* = 28), right part to LMMs including outliers (*N* = 30).

	*N* = 28				*N* = 30			
Predictors	Estimate *β*	*SE*	*t*	*p*	Estimate *β*	*SE*	*t*	*p*
Logarithm of fixation duration 0–5000 ms								
Intercept	5.55	0.05	**115.95**	<0.001	5.55	0.05	**112.12**	<0.001
E_ON_	−0.01	0.02	−0.63	0.526	−0.04	0.02	−1.90	0.058
Δ_EHR_	−0.06	0.02	−**2.93**	0.003	−0.04	0.02	−**2.60**	0.009
Logarithm of fixation duration 0–2000 ms								
Intercept	5.55	0.05	**112.37**	<0.001	5.55	0.05	**120.01**	<0.001
E_ON_	−0.02	0.02	−1.09	0.276	−0.04	0.02	−**2.21**	0.027
Δ_EHR_	−0.06	0.02	−**2.75**	0.006	−0.05	0.02	−**2.68**	0.007

## Discussion

The main aim of this study was to test if individual central bias in scene viewing—when the head is fixed—is related to individual head-movement propensity. Despite establishing measures of either property, we did not find robust evidence for a connection between the two. We found, however, a relation of head-movement propensity and fixation duration—participants with a larger head contribution (Δ_EHR_) in the eye-head-range (EHR) tended to make shorter fixations in scene viewing. The size of the eye-only-range (EOR) characterized by the head-onset threshold (E_ON_) did not influence fixation duration in scene viewing.

Because our experiment was not originally designed to compare fixation durations in scene viewing to head-movement propensity, we can only speculate about potential reasons for the observed relation. Possibly, moving the head rather than the eyes leads to slower shifts of gaze in the real world, which are then compensated by shorter fixation durations. Alternatively, a larger head-movement propensity could be related to more exploratory behavior, which, in turn, with a constrained head, leads to more eye movements when viewing duration is fixed. This interpretation would suggest that fixation durations in scene viewing themselves depend on whether the head is constrained or not, which is contrary to observation (Backhaus & Engbert, 2024), though it should be noted that these authors do not differentiate between eye, head and body contributions to gaze shifts. To test these hypotheses, one may analyze scene viewing with an unconstrained head and possibly different image sizes (note that in our present head-free setting, fixation durations are mainly determined by the rhythm of the task). It should be noted, however, that fixation duration in scene viewing is strongly influenced by the task (Backhaus et al., 2020; Backhaus & Engbert, 2024; Nuthmann, 2017) and image content (Nuthmann, 2017) and in general shortens with unconstrained head (Haskins et al., 2020). The extent to which inter-individual variations add to this variability and how much they are explained by head-movement propensity are also a promising issues for further research, especially given the renewed emphasis on inter-individual differences in scene viewing (De Haas et al., 2019).

Another possible explanation could be related to the fact that more eccentric fixation positions within the eyes' orbital range are usually fixated for shorter durations (Burlingham et al., 2024; Fuller, 1992a) and in extreme cases can cause discomfort and strain. Individuals with high head-movement propensity (that typically “avoid” eccentric eye orientations) might show an even stronger trade-off between orbital eccentricity and fixation duration when under head-fixed conditions.

For the scene-viewing task, we instructed the participants merely “to study the images carefully.” Although this is a common approach in free viewing of natural scenes, it might be argued that it is different from the task in the peripheral-target task, which may be considered more like search. The impact of task on eye-movement patterns is well-established for scene viewing (e.g., Buswell, 1935; Castelhano, Mack, & Henderson, 2009; Yarbus, 1967). Task dependence extends to the central bias (Backhaus et al., 2020; Backhaus & Engbert, 2024; Tatler, 2007) and to other scene-viewing biases that are independent of the current stimulus, such as the extent of pseudoneglect (Nuthmann & Matthias, 2014). Backhaus and Engbert observed effects of task *and* of body posture (standing with free head vs. sitting with constrained head) on central bias, but—crucially—no interaction between these factors. Because the angular subtense of their display (a projection in 2.7m distance subtending 40.6 × 20.1 dva) in the horizontal was similar to our larger scenes (38.2 × 28.7 dva), this leaves us confident that putative head-movement propensity effects would not be shadowed by the exact choice of task in scene viewing. In turn, the task has also a profound impact on eye-head-coordination (e.g., Bischof, Anderson, & Kingstone, 2023; Hu, Bulling, Li, & Wang, 2023; Proudlock & Gottlob, 2007). It might therefore be argued that our measures of head-movement propensity would have differed, if we chose a different task or more relevant (or salient) peripheral stimuli in the peripheral-target task. While the effect of different stimuli, postures (e.g., standing vs. walking) and tasks are interesting issues for future research, the fact that we observe similar results for our exogeneous and our endogenous task leave us confident that the variability and the position of a given individual within this variability is faithfully captured by our approach. In addition, the “task-driven” motivation for head movements and the need to confirm via button press that the target was found in the peripheral-target-task could have led to a more pronounced head-movement propensity than a participant would have during free exploration. Conversely, it could be argued that the need to immediately look back to the center after having looked at the peripheral target reduces the usage of the head. The former assertion is in line with some participants noting a feeling of “more than usual” head-movement usage. In sum, although we cannot full exclude that our choice of tasks reduced the likelihood to find the hypothesized relation, our results suggest that there is no trivial robust relation between the propensity to move one's head and the central bias in scene viewing.

In our study we primarily examined the spatial relation of eye and head position on final gaze-target alignment that did not differ between exogenous and endogenous presentation mode. There is also a spatiotemporal component in eye-head-coordination. For example, in context of driving simulation, Doshi & Trivedi (2012) found a difference in eye-head-dynamic for exogenous and endogenous attention shifts. For endogenous attention shifts the head moved before the eyes; for exogenous shifts eye movements started first. In our data we did not find such differences. However, saccadic eye movements started somewhat later in the endogenous task, which—in our case—is likely explained by the additional time needed to read and process the target information as compared to a salient exogenous cue.

Although task-related (top-down) effects in scene viewing can dominate over stimulus-related (bottom-up) factors from the first saccade onwards (Einhäuser, Rutishauser, & Koch, 2008), whether the first saccade itself is top-down-driven often depends on its latency (Anderson, Ort, Kruijne, Meeter, & Donk, 2015) as does the trade-off between low- and high-level features (Schütt, Rothkegel, Trukenbrod, Engbert, & Wichmann, 2019). In line with this, Rothkegel and colleagues (2017) find that the central bias of the fixation following the first saccade after image onset (“second fixation” in their terms) is enlarged (i.e., its distance from the center is reduced) for short latencies, an effect we confirm in our data. However, we do not find any relation of head-movement propensity to central bias, even if only this fixation is considered and its latency is taken into account. Moreover, while central bias decreases over time (consistent with, e.g., Rothkegel et al., 2017), we do not find any temporal evolution of its relation to our measures of head-movement propensity. Importantly, our positive finding of a relation between fixation duration and head-movement propensity persists, if analysis is restricted to an early phase of scene viewing, during which Backhaus & Engbert (2024) did not find an effect of body posture (standing vs. sitting) on fixation duration (and no interaction of body posture with task). This leaves us confident that our result, which makes an argument about consistency *within* individuals, is not contingent on the fact that head-movement propensity is measured in a task highly distinct from the scene viewing task. In fact, using very distinct tasks strengthens the argument that the observed relation is truly related to a characteristic of the individual rather than to our specific choice of tasks.

Compared to other image properties, relatively little research has addressed the role of image size in the control of eye movements, the guidance of attention or visual perception in general. Although natural scenes do not have an intrinsic scale on average (which is a consequence of their 1/f amplitude spectrum, Field, 1987), individual scenes are often structured by the size of objects and their relation to the scene. Our result that the central bias, which is one of the most important factors contributing to gaze allocation in free viewing (Nuthmann et al., 2017; Tatler, 2007), is largely independent of presentation size is therefore reassuring with respect to the comparability of different scene-viewing studies. It is also in line with Clarke & Tatler's (2014) survey that found the parameters of central bias, when scaled with respect to image size, to be remarkably consistent across various studies. This consistency of central bias across scene size is somewhat remarkable, given that several eye-movement parameters scale with image size, including fixation duration, number of fixations and saccade amplitude (Gameiro, Kaspar, König, Nordholt, & König, 2017; Otero-Millan, Macknik, Langston, & Martinez-Conde, 2013; Von Wartburg et al., 2007), findings we replicated in our present study.

On a more general level, both the null-effect regarding the relation of head-movement propensity and central bias as well as the consistency of central bias relative to image size across image scales, clearly point to the fact that central bias is – albeit highly idiosyncratic – not primarily driven by oculomotor constraints and preferences, but by active scene-viewing processes. This is again consistent with the data of Tatler (2007), who showed an independence of the central bias from oculomotor bias and also image features in free scene viewing. One could argue that this central bias comes from a more screen-related reference frame than scene-related. When parting a scene in quadrants compared to showing it as a single image, fixation distributions shift from screen-centered to quadrant-centered (Stainer, Scott-Brown, & Tatler, 2013). This effect is strongest, when the semantics of the scene as a whole get violated by shuffling quadrant positions. On an even more abstract level, it is plausible that during scene viewing, central bias is just a specific case of the viewing bias towards the object center (PVL; Nuthmann & Henderson, 2010), where the “object” in this case is the full scene. However, it is also obvious that the size independence of central bias must break down at one point, the latest when the image size is such that it would exceed the limit of the oculomotor range, and the head needs to be used in scene viewing as well. Exploring scene-viewing behavior with large stimuli or unconstrained head may therefore provide additional insight to link scene-viewing data from controlled laboratory experiments to viewing behavior in the “wild.”
